# Loss of Cathepsin Z enhances pro-inflammatory macrophage responses and promotes tissue regeneration

**DOI:** 10.1242/dmm.052520

**Published:** 2026-04-29

**Authors:** Thais Sibioni Berti Bastos, Catherine A. Loynes, Zoë C. Speirs, Jordana Dinorá de Lima, Leonel Witckosk Junior, André Guilherme Portela de Paula, Andressa Pacheco Czaikovski, Rebeca Bosso Santos Luz, Lais Cavalieri Paredes, Mia Norris, Gillian S. Tomlinson, Wanderson Duarte da Rocha, Stephen A. Renshaw, Philip M. Elks, Tarcio Teodoro Braga

**Affiliations:** ^1^ Basic Pathology Department, Biological Sciences Sector, Federal University of Paraná, Curitiba 81530-000, Brazil; ^2^ Bateson Centre for Disease Mechanisms and Division of Clinical Medicine, School of Medicine and Population Health, University of Sheffield, Sheffield S10 2TN, UK; ^3^ Biological Sciences Institute IV, University of São Paulo, São Paulo 05508-000, Brazil; ^4^ Biochemistry Department, Biological Sciences Sector, Federal University of Paraná, Curitiba 81530-000, Brazil

**Keywords:** Zebrafish, Macrophages, Regeneration, Fibrosis

## Abstract

The plasticity of macrophages is well documented, with fundamental roles in modulating inflammation and promoting tissue repair, notably aiming to maintain homeostasis in multicellular organisms. However, the precise factors that regulate their polarization remain poorly understood. Cathepsin Z (*CTSZ*) encodes an enzyme highly expressed in macrophages and involved in various processes, such as migration, maturation and signal transduction, but its roles in regeneration are not described. Therefore, we used zebrafish models to investigate the roles of *ctsz* in macrophage polarization and regeneration in the context of sterile inflammation induced by caudal fin transection. CRISPR/Cas9-mediated knockdown of *ctsz* led to higher pro-inflammatory *tnfα^+^* macrophages than in control animals following injury (24-48 h post-injury), as well as accelerated regenerated area. Further studies in this field could prove valuable for the development of pharmacological approaches for chronic diseases characterized by impaired tissue regeneration, such as liver fibrosis or autoimmune diseases, in which dysregulated inflammation and regeneration play critical roles.

## Teaser

**Summary:** Loss of CtsZ promotes a pro-inflammatory macrophage response that accelerates fin regeneration in zebrafish, highlighting *CTSZ* as a potential therapeutic target for tissue repair post injury.

## INTRODUCTION

Wound healing is a complex biological process involving three overlapping phases (inflammation, proliferation and remodelling of the injured tissue), and macrophages actively participate in all these phases (Krzyszczyk et al., 2018). While inflammation is essential for maintaining homeostasis, its dysregulation can lead to chronic conditions that contribute to diseases such as fibrosis, autoimmune disorders and cancer (Gurevich et al., 2018; Rodríguez-Morales and Franklin, 2023). In addition, diseases such as type 2 diabetes, atherosclerosis and inflammatory bowel disease, for example, are characterized by chronic inflammatory processes (Balde et al., 2024; Yang et al., 2024). Macrophages are central players in inflammation, with distinct phenotypes contributing to different stages of the inflammatory response. Pro-inflammatory (M1-like) macrophages promote inflammatory responses, whereas anti-inflammatory (M2-like) macrophages facilitate the resolution of inflammation and tissue repair (Rodríguez-Morales and Franklin, 2023; Yang et al., 2024). Although this binary classification is widely used, it oversimplifies macrophage heterogeneity that occurs *in vivo*; however, it remains useful for studying extreme polarization states (Xue et al., 2024). Given their crucial role in regulating homeostasis and their involvement in various pathologies, macrophages are promising therapeutic targets for wound healing (Ginhoux et al., 2016).

Dysfunction in alternatively activated macrophages (typically M2-like) has been linked to increased extracellular matrix (ECM) deposition, resulting in tissue fibrosis (Mosser and Edwards, 2008). Modulating the factors responsible for M2 macrophage polarization may, therefore, offer a therapeutic strategy to mitigate fibrosis progression (Hu et al., 2023). Despite the well-documented plasticity of macrophages, the precise factors regulating their polarization remain poorly understood (Novak and Koh, 2013). Moreover, a more detailed characterization of distinct macrophage populations is essential to advance our understanding of disease aetiology, alleviate pathological inflammation, promote tissue regeneration, and uncover the complex interactions between innate and adaptive immunity (Mosser and Edwards, 2008; Xue et al., 2024).

*In vivo* genetically modified animal models are crucial for elucidating the biological functions of specific genes within a complex organism, a perspective unattainable through cell culture alone (Chang and Grieder, 2024; Soufizadeh et al., 2024). Mouse models have been instrumental in such studies, but *in vivo* imaging of inflammatory processes is challenging in mouse without surgical procedures, and manipulation of multiple genes is expensive (Dawson et al., 2021; Lehman and Stairs, 2015). The zebrafish (*Danio rerio*), with its genetic tractability and translational relevance, is a powerful model for such studies, which are fundamental for identifying potential therapeutic targets (Fujii et al., 2024; Howe et al., 2013). Here, we used the unique advantages that zebrafish embryos offer, including near-transparent larvae allowing *in vivo* imaging, immune cell transgenic lines and ease of CRISPR/Cas9 F0 knockdown. The zebrafish caudal fin amputation model (Renshaw et al., 2006) has proven particularly suitable for studying the inflammatory process, from its onset to resolution, subsequently leading to tissue regeneration. Unlike humans, zebrafish possess a remarkable ability to regenerate complex tissues and organs, such as the heart, brain and fins (Jimenez Gonzalez et al., 2023; Robertson and Huttenlocher, 2022). Moreover, macrophages play a pivotal role in caudal fin regeneration, yet the molecular mechanisms by which these cells mediate signalling remain poorly elucidated (Nguyen-Chi et al., 2017; Tsarouchas et al., 2018). In adult zebrafish, macrophage ablation significantly reduces regeneration rates, while their absence during tissue growth alters the pattern of regenerated tissue. This suggests that distinct macrophage populations coordinate the regeneration of caudal fin tissues (Nguyen-Chi et al., 2017; Petrie et al., 2014).

Among the molecular players involved in macrophage biology, cathepsins have emerged as critical regulators of immune cell function. In humans, cathepsin Z (CTSZ), a lysosomal cysteine protease with carboxypeptidase activity, is predominantly expressed in immune cells such as monocytes, macrophages, dendritic cells and tumour-associated macrophages (TAMs) (Garin et al., 2001; Journet et al., 2000; Szulc-Dąbrowska et al., 2020). This protease regulates processes such as migration, adhesion, proliferation, maturation, phagocytosis and signal transduction (Fonović et al., 2017). Some recent studies have observed overexpression of *CTSZ* in macrophages infiltrating carcinoma, suggesting that it may serve as a specific macrophage marker and potentially as a prognostic and target biomarker (Zhang et al., 2022).

In order to identify novel regulators of anti-inflammatory macrophage phenotypes during regeneration, we performed bulk RNA-sequencing (RNAseq) analysis of existing human and zebrafish datasets. From this analysis, we selected *ctsz* alongside two other potential anti-inflammatory macrophages genes: *P1, F-box associated domain containing* (*nccrp1*) and *osteoclast stimulatory transmembrane protein* (*ocstamp*)*.* Although less well characterized in the context of macrophage biology, *nccrp1* has been implicated in innate immune responses (Huang et al., 2024), and *ocstamp* is known to be involved in cell fusion processes, including those in macrophage-derived osteoclasts (Yao et al., 2021). These genes were investigated for their potential roles in modulating macrophage phenotypes during regeneration. Among these, only *ctsz* mutations significantly altered both the macrophage polarization and the regenerative process; therefore, we propose that Ctsz functions as a modulator of the macrophage inflammatory response following injury, influencing regenerative outcomes.

## RESULTS

### Identification of candidate genes as markers for anti-inflammatory macrophages

Publicly available RNAseq datasets were interrogated to identify candidate genes for anti-inflammatory macrophages in zebrafish larvae. To identify genes that could be translated to human models, an RNAseq dataset from polarized human monocyte-derived macrophages (MDMs) was compared to a dataset from fluorescence-activated cell sorting-sorted larval zebrafish *Mycobacterium marinum* (*Mm*)-infected macrophages (Baidžajevas et al., 2020; Rougeot et al., 2019). MDMs were isolated from eight donors and polarized towards M1 or M2 *in vitro*, providing a comprehensive transcriptome profile of human polarized macrophages (Baidžajevas et al., 2020). The zebrafish dataset is the only RNAseq profile available of isolated larval macrophages, allowing interrogation of macrophage-specific gene expression, both under resting conditions and following immune challenge with *Mm* (Rougeot et al., 2019). Furthermore, *Mm* challenge is known to modulate macrophages towards an M2-like phenotype (Cronan et al., 2016, 2021; Rougeot et al., 2019), thus increasing the likelihood of identifying potential anti-inflammatory macrophage marker genes that may also be present in M2-polarized MDMs.

**Fig. 1.**
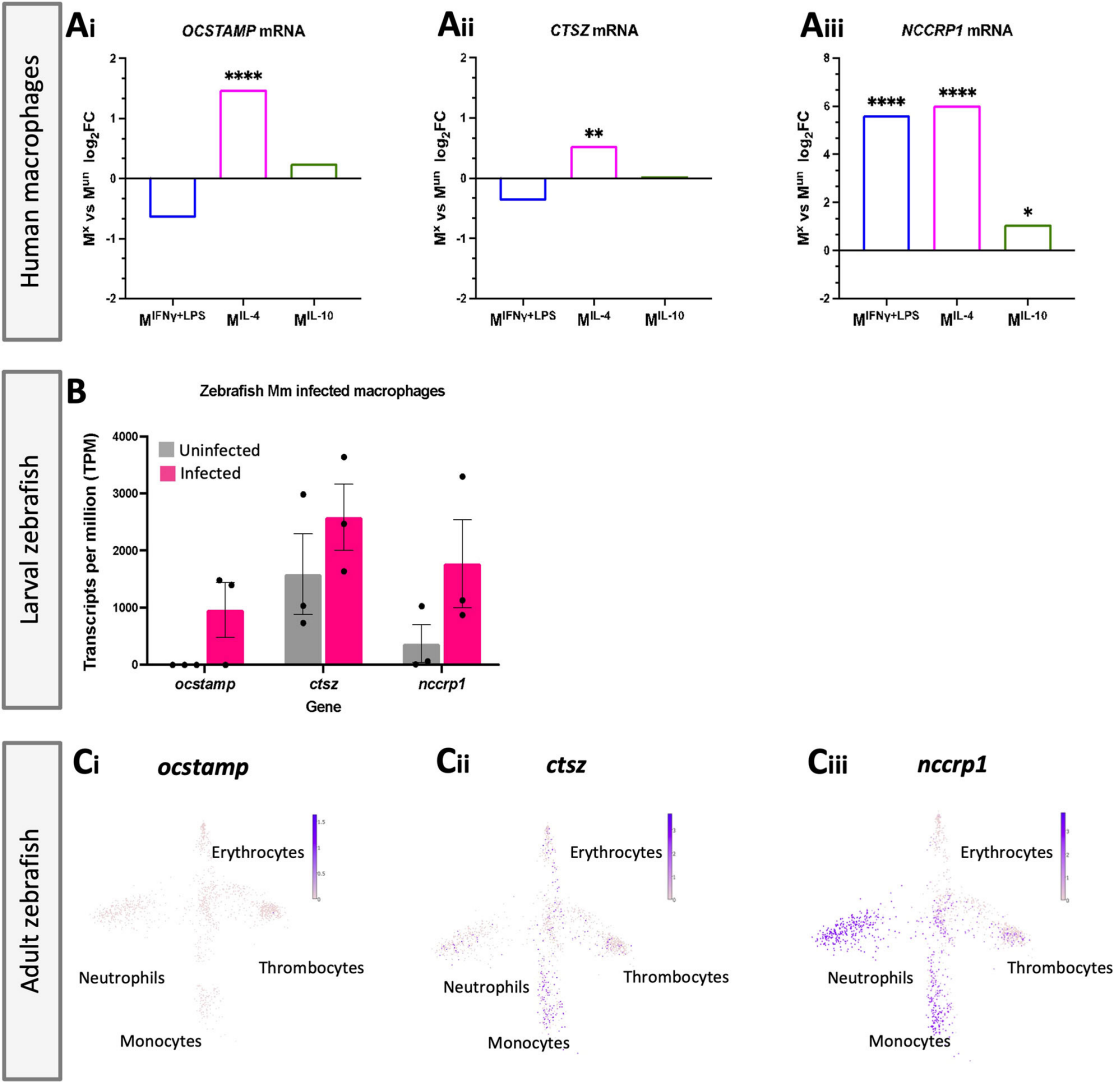
**Comparison between RNA-sequencing (RNAseq) datasets obtained from human polarized macrophages and zebrafish *Mycobacterium marinum* (*Mm*)-infected macrophages identified three candidate genes for anti-inflammatory macrophages.** (Ai-iii) RNAseq of human macrophages polarized *in vitro* from eight donors (Baidžajevas et al., 2020). M1 macrophages were polarized with IFNγ+LPS (M^IFN+LPS^); M2-like macrophages were polarized with either IL-4 (M^IL-4^) or IL-10 (M^IL-10^). Polarized macrophages were compared to unpolarized ones. Expression of *OCSTAMP* (Ai), *CTSZ* (Aii) and *NCCRP1* (Aiii) in polarized macrophages compared to unpolarized macrophages is shown. To comply with the ethical approval of the original study, data are presented as mean values only, as individual biological replicate data were not available for publication. FC, fold change. (B) RNAseq of 5 days post-infection isolated zebrafish macrophages following *Mm* infection (Rougeot et al., 2019). For this analysis, 20 infected and uninfected cells were fluorescence-activated cell sorting (FACS) purified (*n*=3 independent repeats) and used for cDNA synthesis, with resulting cDNA amplified for RNAseq. Data are means from three replicates; error bars depict s.e.m. No statistical differences were identified using an unpaired two-tailed *t*-test. (Ci-iii) Expression levels of these genes in four zebrafish blood cell types: erythrocytes, neutrophils, monocytes and thrombocytes. The atlas was generated with single-cell RNAseq (scRNAseq) of FACS-purified kidney-derived blood cells from eight transgenic and one non-transgenic fish, obtained from BASiCz – Blood Atlas of Single Cells in zebrafish. Interestingly, there is very little expression of *ocstamp* in adult zebrafish leukocyte populations (Ci). Expression of *ctsz* also shows minimal expression in leukocyte populations, but it is most prominently expressed in monocytes (Cii). This suggests that *ocstamp* and *ctsz* could be strong candidates for anti-inflammatory macrophages as low expression levels in unchallenged fish would be expected. However, *nccrp1* is more widely expressed, particularly in both neutrophil and monocyte adult leukocyte populations (Ciii), suggesting that this gene may not be specific enough to be an anti-inflammatory macrophage marker. Each arm of the diagram indicates a haematopoietic cell population, and each dot represents separate single-cell RNAseq repeats performed. Populations are labelled on the image. Deeper colour indicates higher gene expression in that population. *False discovery rate (FDR)<0.05; **FDR<0.01; ****FDR<0.0001.

Genes upregulated in MDMs polarized towards an anti-inflammatory phenotype (IL-4 or IL-10 stimulated) and larval zebrafish *Mm*-infected macrophages were compared, and three candidate genes were identified in both datasets – *ocstamp*, *ctsz* and *nccrp1* – based on differential gene expression after infection (Fig. 1). In addition to being upregulated in MDMs polarized with IL-4 [1.48 log_2_fold change (FC); Fig. 1Ai], *OCSTAMP* was found to be significantly downregulated in MDMs polarized towards a pro-inflammatory phenotype [IFNγ and lipopolysaccharide (LPS) stimulated; −0.65 log_2_FC; Fig. 1Ai], and elevated *ocstamp* transcript levels were identified in zebrafish *Mm*-infected macrophages compared to uninfected macrophages [960.3 transcripts per kilobase million (TPM) compared to 2 TPM; Fig. 1B]. Similarly, *CTSZ* was downregulated in M1-polarized MDMs (−0.38 log_2_FC) and significantly elevated when stimulated with IL-4 (0.54 log_2_FC; Fig. 1Aii). *ctsz* transcript levels were high in infected and uninfected zebrafish macrophages (2586 TPM compared to 1587.3 TPM; Fig. 1B). Finally, *NCCRP1* was significantly upregulated in MDMs stimulated with IL-4 and IL-10 (6.01 log_2_FC and 1.08 log_2_FC, respectively), and also significantly upregulated in MDMs stimulated with IFNγ and LPS (5.62 log_2_FC; Fig. 1Aiii). This gene was included as a candidate anti-inflammatory marker as *nccrp1* was upregulated in zebrafish infected macrophages (1770 TPM compared to 367.7 TPM; Fig. 1B).

### Candidate gene expression in larval zebrafish haematopoietic populations indicates macrophage specificity

**Fig. 2.**
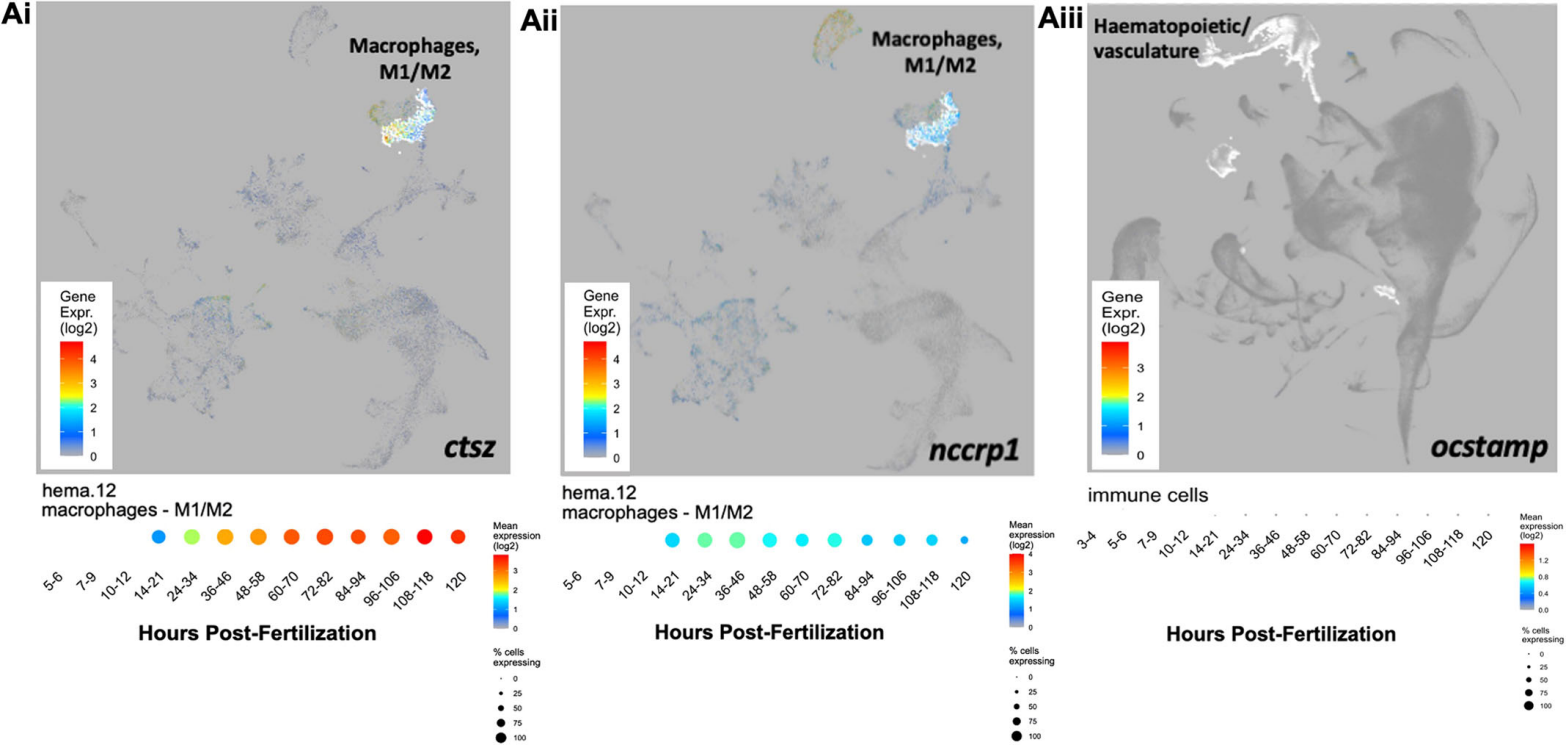
**Under resting conditions, *ctsz* and *nccrp1* are expressed in larval zebrafish macrophages, with very low expression levels of *ocstamp* in haematopoietic populations.** (Ai-iii) Uniform manifold approximation and projections (UMAPs) of the larval haematopoietic/vasculature cluster: *nccrp1* in the macrophage cluster within the haematopoietic system (Ai); *ocstamp* was not detected in the haematopoietic system (Aii); and *ctsz* expression across development suggests highest expression at ∼4.5 days post-fertilization (dpf) (Aiii). Timeline below UMAPs indicates gene expression across larval development up to 5 dpf.

To further determine expression levels of these candidate genes in zebrafish larvae, haematopoietic expression of the genes was assessed using Daniocell (Sur et al., 2023). When macrophage clusters were assessed, *nccrp1* could be identified in the haematopoietic uniform manifold approximation and projection (UMAP), with macrophage expression of *nccrp1* across development [0.5-5 days post-fertilization (dpf)] remaining low, suggesting that it is not part of a developmental wave of gene expression changes and that it may be specific enough to label anti-inflammatory macrophages *in vivo* (Fig. 2Ai). Expression of *ocstamp* was not detected in the haematopoietic system; thus, no UMAP specifically of the haematopoietic system and macrophage cluster could be generated (Fig. 2Aii). Expression of *ctsz* across development from 3 h post-fertilization (hpf; ∼1000 cells) up to 5 dpf suggested highest expression at ∼4.5 dpf, before starting to fall again (Fig. 2Aiii).

### Zebrafish candidate genes share homology with human orthologues

**Fig. 3.**
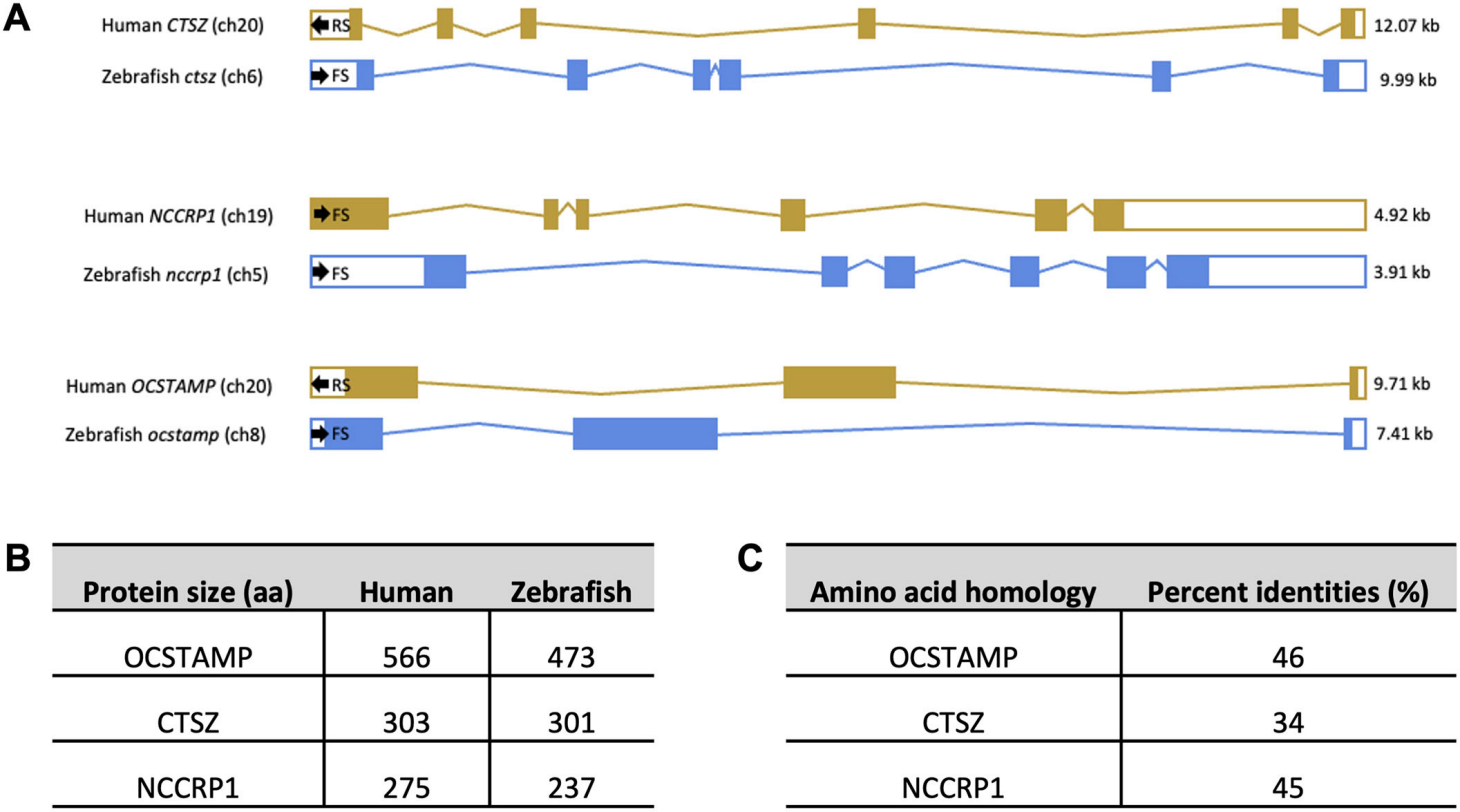
**Zebrafish gene organization of candidate genes share high homology with human orthologues.** (A) Gene organization of human genes (yellow) and zebrafish gene (blue) orthologues share very similar exon numbers and coding exons. Exon maps were generated from the Ensembl database. Filled sections indicate coding exons; empty sections indicate non-coding exons. Chromosome number (ch), strand direction (FS, forward strand; RS, reverse strand) and gene size in kilobases (kb) are stated on exon maps. (B) Human and zebrafish protein sizes (aa, amino acids) of *CTSZ* and *NCCRP1* encode proteins of very similar sizes. Zebrafish *ocstamp* is smaller than the human counterpart. Protein size was determined using the Ensembl database. (C) NCBI Blast Global Align comparing zebrafish sequences to human sequences identified high amino acid homology, depicted as percentage identities.

Zebrafish genes often have multiple isoforms of genes due to a genome duplication event ∼350 million years ago (Hoegg et al., 2004); however, these three candidate genes (*ctsz*, *nccrp1* and *ocstamp*) have only a single isoform, identified from the Ensembl database. Comparison of gene organization of zebrafish *ctsz* (ENSDARG00000043081), *nccrp1* (ENSDARG00000035326) and *ocstamp* (ENSDARG00000022139) to human orthologues found high homology, with all three genes having the same number of exons as human counterparts. Human *CTSZ* and zebrafish *ctsz* share six exons. Although both have a small non-coding region at the start of exon 1, zebrafish *ctsz* has a larger non-coding region at the end of exon 6 compared to the human orthologue. Human *CTSZ* is larger than zebrafish *ctsz* (12.07 kb compared to 9.99 kb), and it is located on the reverse strand of chromosome 20, whereas zebrafish *ctsz* is located on the forward strand of chromosome 6 (Fig. 3A). Zebrafish *ctsz* shares synteny with human *CTSZ*, with *gnas*/*GNAS*, *nelfcd*/*NELFCD* and *npepl1*/*NPEPL1* genes in close proximity in both zebrafish and human genomes. Similarly, human *NCCRP1* and zebrafish *nccrp1* also have six exons, with both genes having a larger non-coding region at the end of exon 6. However, zebrafish *nccrp1* has a larger non-coding region at the start of exon 1. As with the other candidate genes, human *NCCRP1* is slightly larger than the zebrafish orthologue (4.92 kb compared to 3.91 kb), but both zebrafish *nccrp1* and human *NCCRP1* are located on forward chromosome strands (chromosome 5 and 19, respectively) (Fig. 3A). Zebrafish *ocstamp*, like human *OCSTAMP*, has three exons, with small non-coding regions at the beginning of exon 1 and the end of exon 3. Zebrafish *ocstamp* is smaller than the human orthologue (7.41 kb compared to 9.71 kb), and although human *OCSTAMP* is found on the reverse strand of chromosome 20, zebrafish *ocstamp* is located on the forward strand of chromosome 8 (Fig. 3A).

The size of the proteins encoded by these candidate genes was assessed next. Zebrafish and human *ctsz*/*CTSZ* and *nccrp1*/*NCCRP1* encode proteins of very similar sizes [Ctsz, 301 amino acids (aa); CTSZ, 303 aa; Nccrp1, 237 aa; NCCRP1, 275 aa], with zebrafish *ctsz* and *nccrp1* having conserved homology of 34% and 45%, respectively, for human orthologues (Fig. 3B,C). Looking at the overall protein size (in aa), zebrafish Ocstamp is smaller than the human counterpart (473 aa compared to 566 aa) (Fig. 3B). By using the NCBI Blast Global Align tool to compare human sequences to zebrafish orthologues, zebrafish *ocstamp* was found to share the highest identity match (46%) of the three candidate genes, indicating conserved amino acid homology (Fig. 3C).

Together, these data identify additional candidate genes, which, following validation, could be used as markers for anti-inflammatory macrophages in zebrafish. Owing to the heterogeneity of the macrophage anti-inflammatory phenotype, this could allow us to further elucidate macrophage signalling. Additionally, the similarities in gene organization between human and zebrafish orthologues highlights a potentially conserved role of these genes between humans and zebrafish, suggesting that these candidate genes could translate to human systems.

### *In situ* hybridization confirms upregulation of *ctsz* expression following injury

**Fig. 4.**
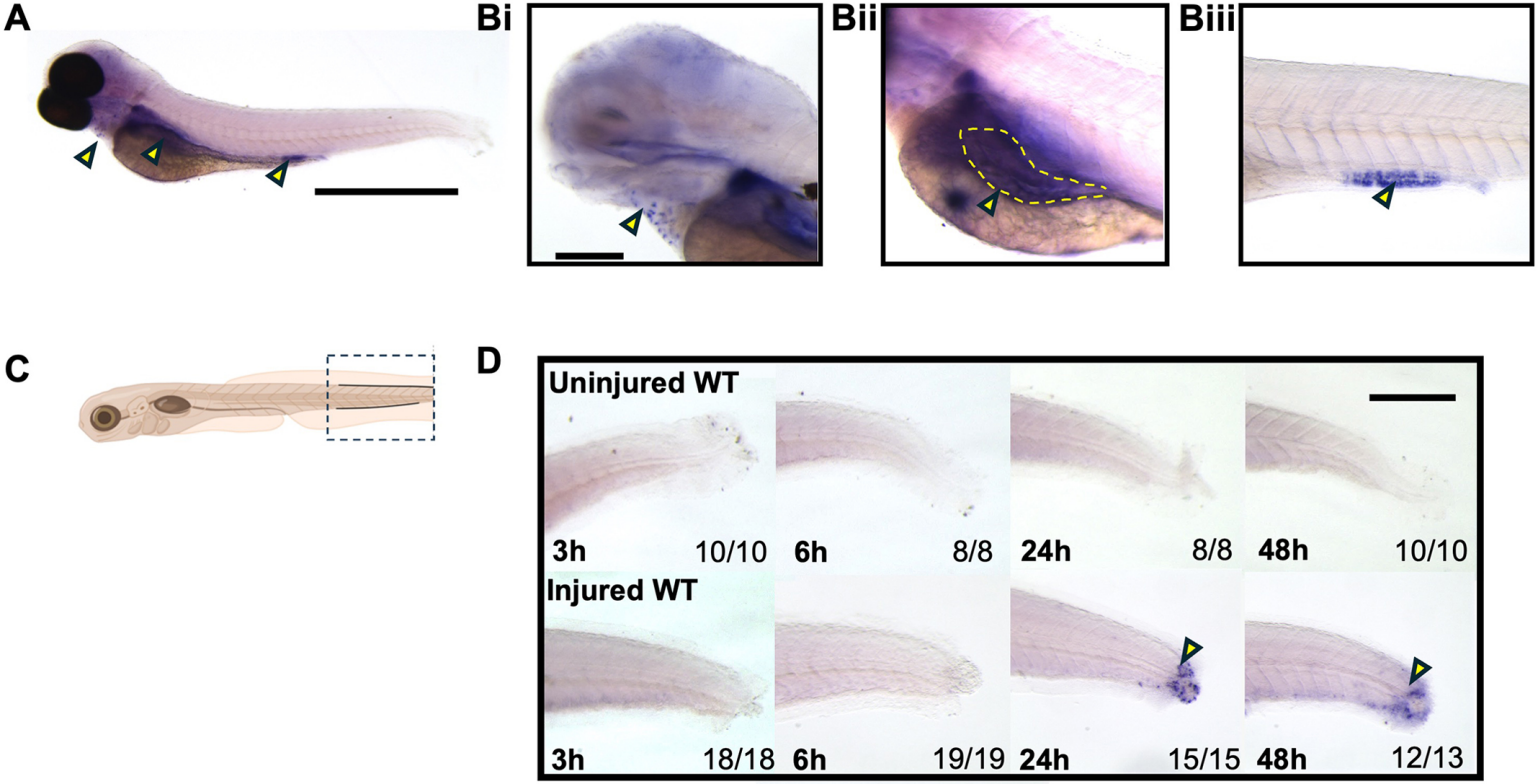
**Wholemount *in situ* hybridization demonstrates *ctsz* expression in cells in the injured tailfin.** (A-Biii) *In situ* hybridization of *ctsz* in unchallenged larvae at 3 dpf (A) highlighting cells overlying the heart cavity (Bi), the intestinal bulb (Bii) and mid-intestine (Biii). (C) Tailfin injury schematic view demonstrating the analysed area in the *in situ* hybridization of *ctsz* experiments. (D) Representative images of *in situ* hybridization of *ctsz* in unstimulated wild-type (WT) (top row) and injured WT (bottom row) larvae at different time points. Numbers at bottom right indicate the number of analysed animals. Yellow arrowheads indicate staining at recruited individual leukocytes. Yellow dashed line outlines the intestinal bulb. Scale bars: 1 mm (A); 500 µm (D); 330 µm (Bi).

Given the *ctsz* expression in monocytes (Fig. 1Cii), in larval zebrafish macrophages (Fig. 2Aiii), its expression modulation through M1- and M2-like inducers (Fig. 1Aii), and the recent discovery that *Ctsz* plays a role in macrophage biology in tuberculosis context (Meade et al., 2025), we decided to further perform *in situ* hybridization of *ctsz* in 3 dpf wild-type (WT) larvae to assess expression in unchallenged larvae and throughout an inflammatory time course, induced by tailfin injury. Unchallenged WT larvae expressed *ctsz* at 3 dpf (Fig. 4A) in cells overlying the heart cavity (Fig. 4Bi). Expression of *ctsz* was also seen in the intestinal bulb (Fig. 4Bii) and mid-intestine epithelium (Fig. 4Biii), where it is known to be involved in protein absorption in lysosome-rich enterocytes (Childers et al., 2025). Following tailfin injury, *ctsz* expression could not be detected at the wound site until 24 h post-injury (hpi) (Fig. 4C,D), when expression was strong within recruited individual leukocytes (yellow arrowheads in Fig. 4D). By 48 hpi, expression was still visible at the wound site but had markedly reduced compared to that at 24 hpi (Fig. 4C,D). These data and time points indicate that these cells are likely to be macrophages, as other leukocytes involved in the inflammatory process, such as neutrophils, accumulate up to 6 hpi and are removed by 24 hpi (Gray et al., 2011).

### Crispants for *ctsz* knockdown demonstrate reduced transcript levels

**Fig. 5.**
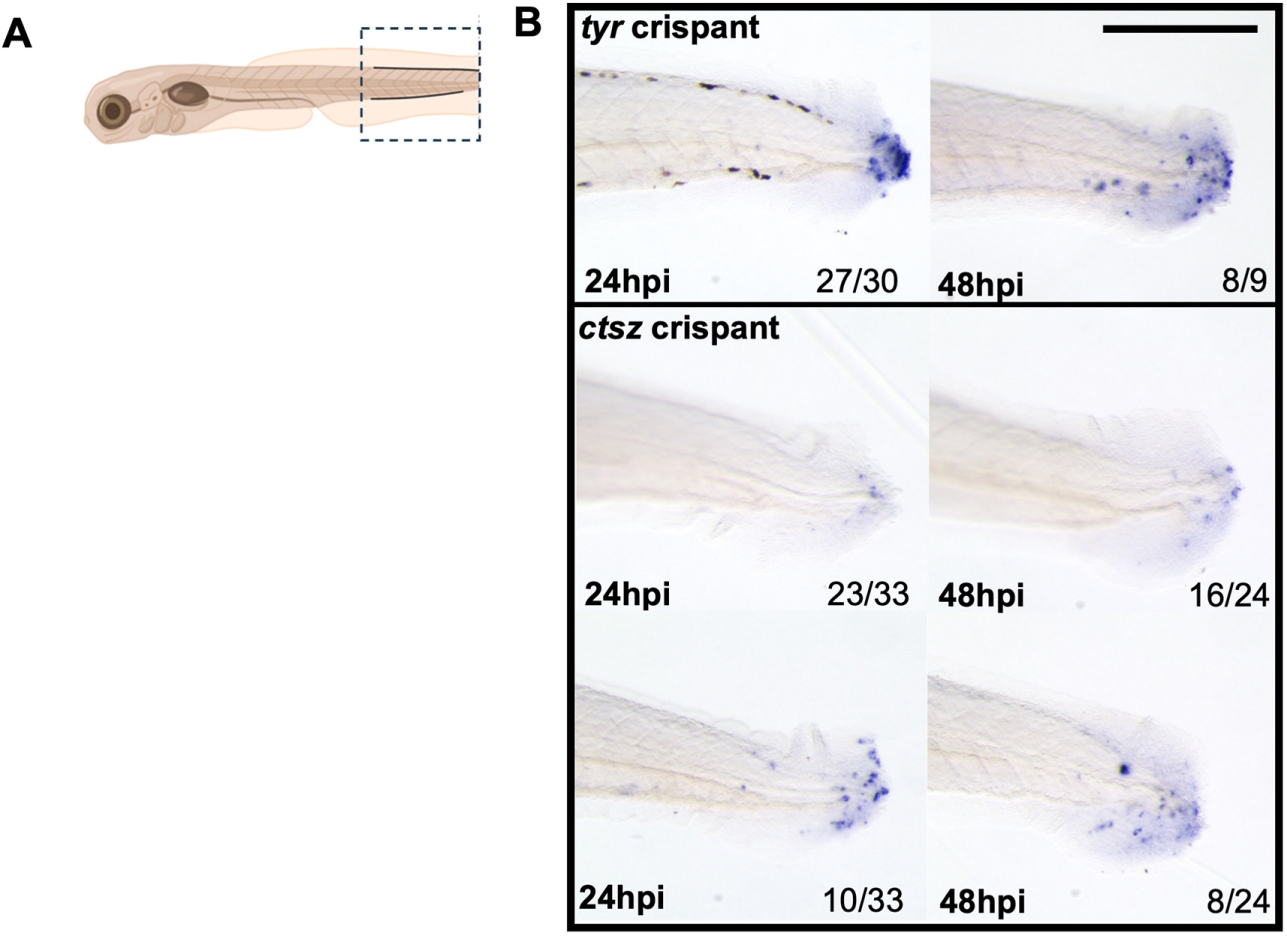
***ctsz in situ* hybridization shows reduced expression in *ctsz* crispant larvae.** (A) Tailfin injury schematic view demonstrating the analysed area in the *in situ* hybridization of *ctsz* experiments. (B) Representative images of *in situ* hybridization of tyrosinase (top row) and *ctsz* (middle and bottom rows) at 24 h post-injury (hpi) (left column) and 48hpi (right column). There were two phenotypes in the *ctsz* knockdown larvae: weak *ctsz* expression (middle row) and a relatively higher expression of *ctsz* at the wound site (lower row) at both 24 and 48 hpi. Numbers at bottom right indicate the numbers with that phenotype and total number of analysed larvae. Scale bar: 500 μm.

These data indicate *ctsz* is an interesting gene that may play a key role in dictating macrophage phenotype. To validate the *ctsz* crispants at the transcript level, *in situ* hybridization was performed in *tyrosinase* (*tyr*) and *ctsz* crispants to assess *ctsz* transcript levels and determine whether nonsense-mediated decay was occurring within these crispant larvae (Fig. 5). All *ctsz* crispants showed reduced *ctsz* expression relative to *tyr* controls following tailfin injury at both 24 and 48 hpi, confirming effective gene knockdown. Within the *ctsz* crispant population, two distinct phenotypes were observed. Sixty-seven percent of larvae exhibited weak *ctsz* expression at both 24 and 48 hpi, with fewer than ten *ctsz*^+^ cells visible at the wound site (Fig. 5B, top row), indicating strong knockdown of gene expression. Thirty percent of the *ctsz* crispant larvae exhibited a higher level of *ctsz* at the wound site, with an average of ∼15 *ctsz*^+^ cells present (Fig. 5B, bottom row), indicating weaker knockdown with potentially functional transcripts produced. All of the *ctsz* knockdown fish, however, had lower *ctsz* expression compared to strong staining in ∼90% of *tyr* control larvae.

### *ctsz* crispant animals exhibit altered macrophage polarization upon fin fold injury

**Fig. 6.**
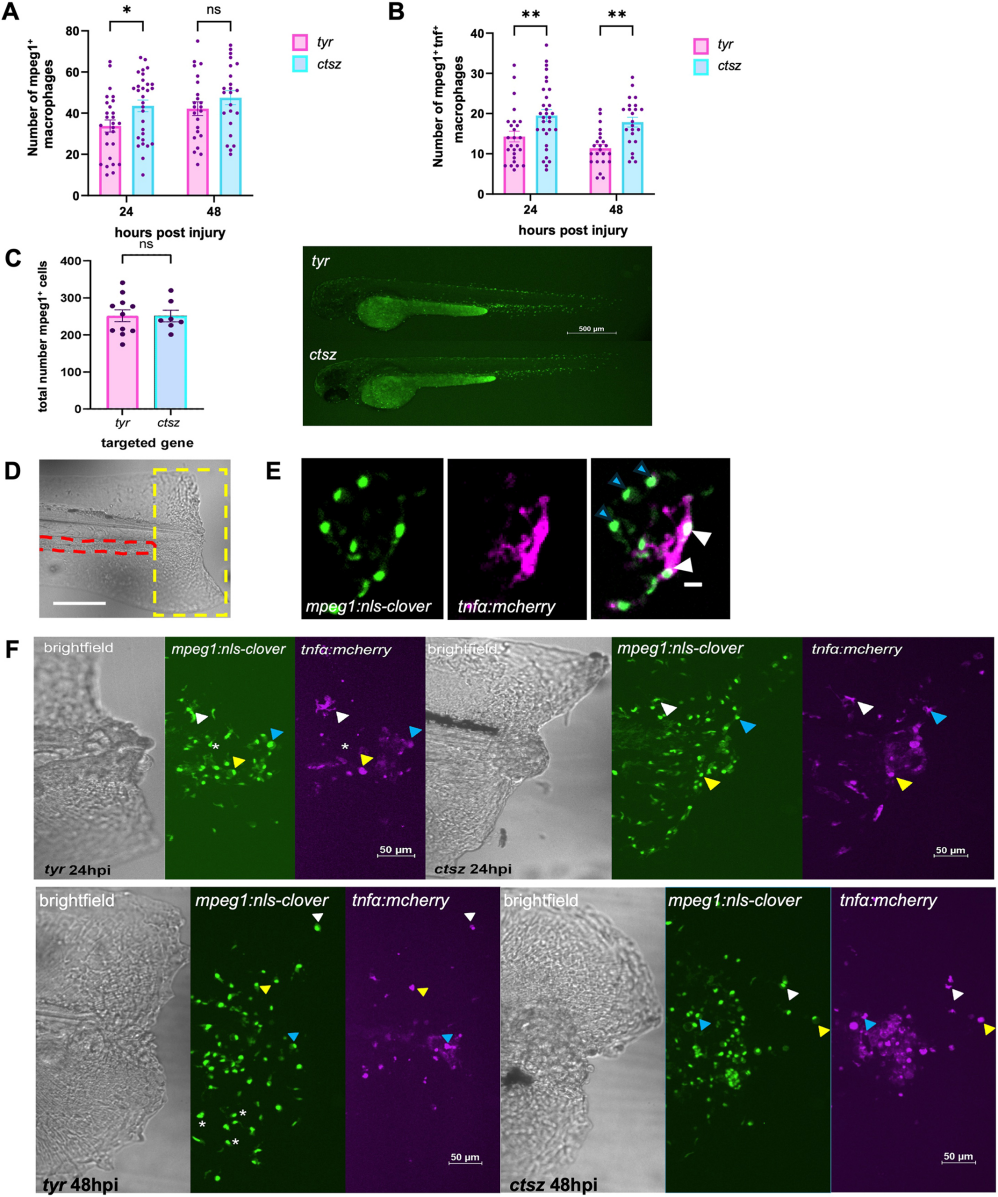
**Evaluation and quantification of total and inflammatory macrophages in *ctsz* crispant larvae.** (A,B) Quantification of the number of total macrophages (*mpeg1^+^tnfα^−^* cells; A) and pro-inflammatory macrophages (*mpeg1^+^tnfα^+^* cells; B) in transgenic *Tg(mpeg1.1:nls-clover/tnfα:mcherry)* tyrosinase control animals (pink) and crispants for the *ctsz* gene (blue) at 24 and 48 hpi. Each point represents a single larva. Data represent three independent repeats; ns, not significant; *P<0.05; **P<0.01 (two-way ANOVA with Bonferroni post-test). (C) Counts of macrophages in unstimulated 2 dpf *tyr* (ten larvae) or *ctsz* (seven larvae) crispants with representative images, analysed by unpaired two-tailed *t*-test. Scale bar: 500 µm. (D) Representative image of the tailfin wound site of *tyr* control and *ctsz* crispant larvae obtained by confocal microscopy. Red dashed line indicates the circulation loop; yellow dashed yellow line indicates the region of the tailfin wound in which macrophages were imaged in subsequent images. Scale bar: 100 µm. (E) Representative images of macrophage (green) and *tnfα* (magenta) expression. Scale bar: 10 µm. (F) Representative images of the caudal inflammatory region, macrophage (green) and *tnfα* (magenta) expression of *tyr* control (top row) and *ctsz* crispant animals (bottom row) at 24 and 48 hpi. White, yellow and blue arrowheads indicate dual expression of *mpeg1* and *tnfα.* Asterisk indicates cells with no *tnfα* expression. Scale bars: 50 µm.

Macrophages participate in all stages of tissue repair, with distinct phenotypes playing specific and critical roles throughout the process (Wynn and Vannella, 2016). During resolution of inflammation, a prerequisite process for tissue remodelling and repair (Sugimoto et al., 2019), bone marrow-derived inflammatory macrophages in mammals and renal-derived inflammatory macrophages in zebrafish often outnumber the tissue-resident macrophages (Davies et al., 2013). Quantifying these inflammatory macrophages is essential for assessing the extent and progression of the inflammatory response. To achieve this, we aimed to quantify recruited macrophages using double-transgenic animals, *Tg(mpeg1.1:nls-clover/tnfα:mcherry).* These animals express nuclear-localized Clover specifically in macrophages, driven by the *mpeg1* (also known as *mpeg1.1*) promoter, with mCherry fluorescent protein under the Tnfα (also known as Tnfa) pro-inflammatory cytokine promoter. This dual labelling allows the differentiation of pro-inflammatory macrophages from other phenotypes, as these cells exhibit overlapping fluorescence (Fig. S1A-E). *ctsz* crispant animals showed a significant increase in macrophage numbers at the wound site at 24 hpi, with numbers remaining high at 48 hpi (Fig. 6A). When analysing only the number of pro-inflammatory macrophages (*mpeg1^+^tnfα^+^* cells), crispant animals exhibited a significantly higher number at both 24 and 48 hpi compared to the control non-crispant group, with these increases not due to an overall increase in total macrophage number in non-wounded *ctsz* crispants (Fig. 6B-F).

We additionally sought to identify any differences in macrophage numbers at the site of tissue regeneration in *nccrp1* and *ocstamp* crispant animals. Using the transgenic zebrafish line *TgBAC(csf1ra:eGFP)sh377*, in which macrophages express green fluorescent protein (GFP), we analysed macrophages based on the fluorescence intensity emitted by these cells. None of the genes knocked down were able to alter the number of macrophages at the injury site (Fig. S2A,B).

### Zebrafish larvae with mutations in *ctsz* exhibit enhanced caudal fin regeneration

**Fig. 7.**
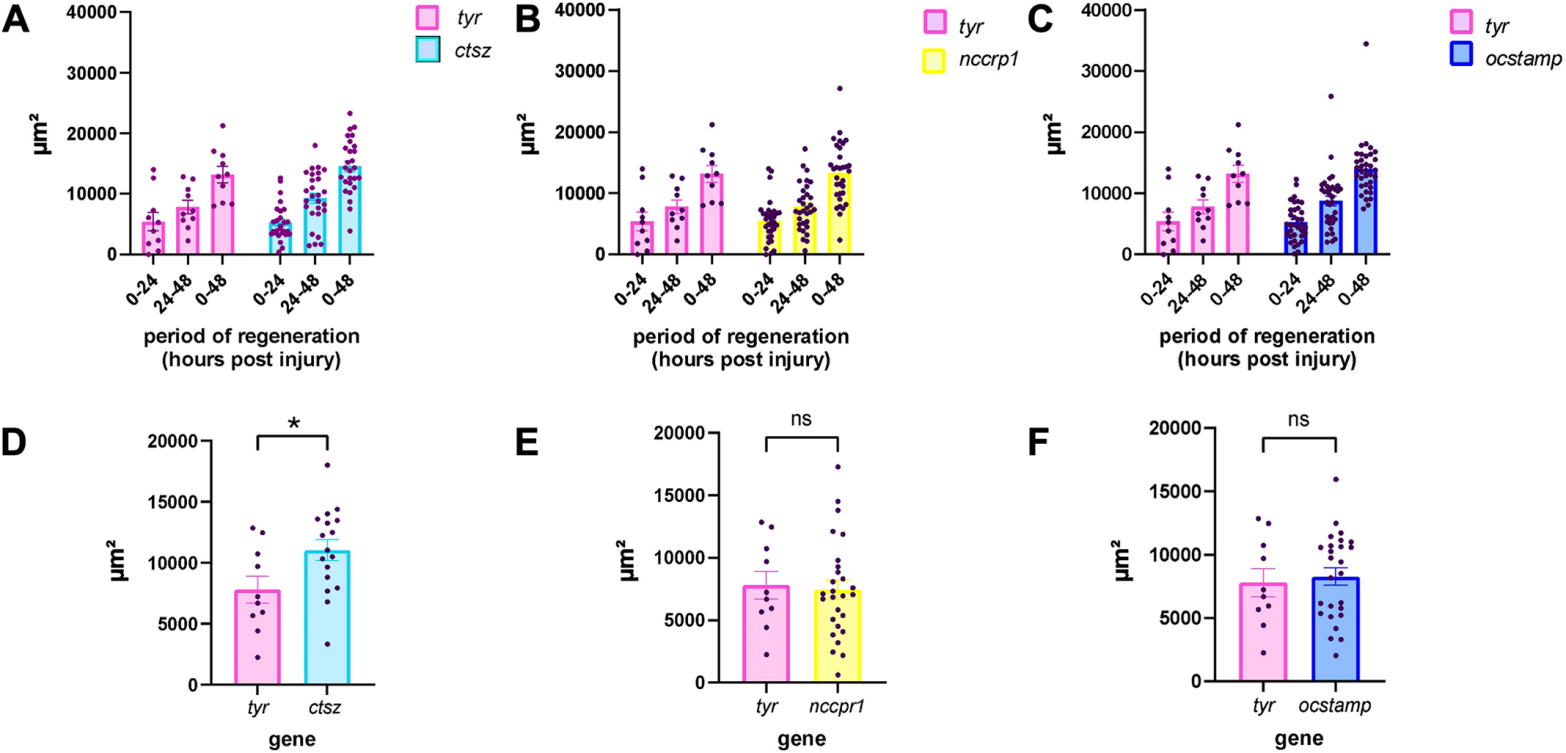
**Regeneration area in crispants for *ctsz*, *nccrp1* and *ocstamp* genes versus that in *tyr* control animals.** (A-C) Regenerated area in crispants for the *ctsz* (A), *nccrp1* (B) and *ocstamp* (C) genes versus that in *tyr* control animals. The normalized regenerated area was calculated for three intervals: regeneration during the first 24 hpi (area at 24 hpi−area at 0 hpi), regeneration during the second 24 hpi (area at 48 hpi−area at 24 hpi) and total regeneration up to 48 hpi (area at 48 hpi−area at 0 hpi). (D) Regeneration area during the second 24 hpi period (area at 48 hpi−area at 24 hpi) of *ctsz* crispant animals (blue) and *tyr* control (pink). (E) Regeneration area during the second 24 hpi of *nccrp1* crispant animals (yellow) and *tyr* control (pink). (F) Regeneration area during the second 24 hpi of *ocstamp* crispant animals (dark blue) and *tyr* control (pink). In D-F, the analysis was performed in crispants for *ctsz* (D), *nccrp1* (E) and *ocstamp* (F) genes only where an edit is detectable on a gel, indicating a significant disruption to the gene. Area is expressed in µm², and each point represents a single animal. Two-way ANOVA with Bonferroni multiple comparison test were applied (ns, not significant; *P<0.05); error bars represent mean±s.d.

To investigate the *in vivo* functional role of the *ctsz*, *nccrp1* and *ocstamp* genes during regeneration, crispant animals were subjected to a sterile inflammatory response followed by tissue regeneration. Caudal fin regeneration was assessed at three time points following fin fold transection: immediately after injury (0 hpi), and at 24 and 48 hpi. When comparing the regenerated area of crispants for the *ctsz*, *nccrp1* and *ocstamp* genes with that of the *tyr* control animals at all time points, we observed that none of the crispants resulted in altered regeneration (Fig. 7A-C). We further assessed the amount of regeneration between 24 and 48 hpi in larvae in which an edit is detectable on a gel, indicating significant disruption to the gene and, potentially, its function. Crispants for the *ctsz* gene demonstrated higher regenerated area than did crispants for the *tyr* gene (Fig. 7D). In contrast, crispants for the *nccrp1* and *ocstamp* genes showed no significant differences from *tyr* control animals (Fig. 7E,F). Altogether, these data suggest that, in addition to a greater number of M1-like cells, knockdown of *ctsz* leads to accelerated regeneration followed by tailfin injury in zebrafish.

## DISCUSSION

This study identified three candidate genes for anti-inflammatory macrophage marker genes (*CTSZ*, *NCCRP1*, and *OCSTAMP*) from M2-polarized human MDMs that have zebrafish orthologues. *CTSZ* mRNA levels have been found to be elevated in M2-like TAMs, suggesting that this gene is conserved in mammalian macrophages polarized towards an anti-inflammatory phenotype (Cui et al., 2021). Currently, no studies have examined the potential role of *NCCRP1* in determining macrophage phenotype, with human *NCCRP1* being associated with squamous epithelium-containing tissues (Kallio et al., 2011). However, its orthologue in several fish species (e.g. zebrafish, Atlantic salmon, Nile tilapia, northern snakehead, yellow croaker) has been associated with non-specific cytotoxic cells, particularly during infection challenge (Hu et al., 2024; Jaso-Friedmann et al., 1997; Niu et al., 2020; Sun et al., 2024; Teng et al., 2023; Wu et al., 2021; Yao et al., 2024). Overexpression of *Ocstamp* has also been found to suppress M1 polarization in RAW264.7 cell culture, and although osteoclasts are a bone marrow-resident cells with phagocytic activity, they are derived from the macrophage lineage (Lee et al., 2021; Yuan et al., 2017).

Using CRISPR/Cas9, we generated deletions in *ctsz*, *nccrp1* and *ocstamp* in zebrafish, achieving mutation efficiencies of 47.6%, 60% and 53%, respectively. Crispants for the *ctsz* gene exhibited a higher number of total recruited macrophages at 24 hpi than did controls, with an increased number of pro-inflammatory macrophages seen at 24 and 48 hpi. Functional analysis revealed that only *ctsz* mutations significantly altered the regenerative response, increasing the regeneration area between 24 and 48 hpi. Mutations in *nccrp1* and *ocstamp*, in turn, did not result in any significant changes in the parameters assessed.

Our findings indicate that *ctsz* plays a regulatory role in macrophage polarization following injury. Its deletion altered the balance between pro-inflammatory and anti-inflammatory macrophages, increasing *tnfα^+^* cells at 24 and 48 hpi in response to fin fold transection. Our data support the hypothesis that *ctsz* may act as an inflammatory modulator, and it is conceivable that *ctsz* plays a role in promoting M2 polarization. Previous studies have demonstrated the kinetics of macrophage recruitment during regeneration, showing that macrophages are rapidly recruited to the injury site and remain present for up to 3 days post-amputation. M1-like macrophages (*mpeg1*^+^/*tnfα*^+^) peak at 6 h post-amputation (hpa), while M2-like macrophages (*mpeg1*^+^/*tnfα*^−^) are present from 6 hpa to 3 days post-amputation, peaking between 24 and 72 hpa (Bohaud et al., 2021; Nguyen-Chi et al., 2017).

It has been shown that early vascular sprouting at the injury site depends on pro-inflammatory macrophages expressing *vegfa* (Gurevich et al., 2018). Additionally, studies have demonstrated that early macrophage ablation impairs fin growth after amputation, whereas late depletion does not affect blastema cell proliferation (Nguyen-Chi et al., 2017). In adult zebrafish, total macrophage ablation using the nitroreductase/metronidazole system has been shown to reduce regeneration at later time points (Petrie et al., 2014). Our study, however, focused on the quantification of the regenerated area, and histological analyses could offer deeper insights into the architecture and organization of the regenerated tissue. Crispants for the *ctsz* gene exhibited a higher proportion of macrophages expressing *tnfα* at 24 and 48 hpi and increased regenerated area. The absence of *ctsz* could have led to an accelerated regenerative process, characterized by a more pronounced pro-inflammatory polarization of macrophages during the first 24 h of the inflammatory response.

In both humans and zebrafish, macrophage migration and stromal remodelling depend on the degradation of the ECM by cathepsins and matrix metalloproteinases (MMPs) (Travnickova et al., 2015). Blocking cathepsin activity has been shown to inhibit macrophage migration in Matrigel and collagen gel assays (Hirakawa et al., 2007). Specific cathepsins – such as cathepsin S, B and L – play key roles in macrophage migration in various disease models, including atherosclerosis, transplant rejection and infection (Christen et al., 2009; Hirakawa et al., 2007; Kitamoto et al., 2007). Moreover, macrophage migration and ECM remodelling have been linked to the expression of MMPs, as demonstrated in studies involving *irf8* mutants (Tsarouchas et al., 2018). CTSZ has been implicated in several inflammatory processes and malignancies (Akkari et al., 2014; Dolenc et al., 2021; Mountford et al., 2020; Nägler et al., 2006). Although its role in health and disease is not fully understood, elevated plasma levels of CTSZ have been observed in patients with poor prognoses following trauma, suggesting its potential as a biomarker for inflammatory disorders (Nägler et al., 2006). A CTSZ mouse mutant has linked cathepsins to breast cancer progression and metastasis and provides the option of further characterization of its role in macrophages in murine disease models (Sevenich et al., 2010). It has also been demonstrated that CTSZ variants in humans and genetically diverse mice mediate tuberculosis disease severity (Meade et al., 2025). Our findings suggest that *ctsz* regulates macrophage polarization dynamics following injury during the regeneration process. Its absence promotes an initial pro-inflammatory response, enhancing early regeneration. This highlights *CTSZ* as a potential therapeutic target for modulating inflammation and tissue regeneration.

## MATERIALS AND METHODS

### Zebrafish husbandry and ethics

To study the inflammatory response of macrophages, we used *TgBAC(csf1ra:eGFP)sh377*, as well as a *Tg(mpeg1.1:nls-clover)sh436* (Elworthy et al., 2023) and *TgBAC(tnfα:mcherry)sh716* cross, known as *Tg(mpeg1.1:nls-clover/tnfα:mcherry)*. Adult zebrafish were maintained in a zebrafish rack system at 28°C with continuous water recirculation and a 14 h/10 h light/dark cycle. Adult fish were paired to obtain embryos.

For experiments conducted in the UK, the zebrafish were raised at the Bateson Centre for Disease Mechanisms, University of Sheffield, in the Biological Services Aquaria approved by the UK Home Office and maintained according to standard protocols. All procedures were carried out on embryos <5 dpf, thus falling outside the scope of the Animals (Scientific Procedures) Act, and followed UK Home Office standards. Adult animals were covered under PPL 9853040C.

For experiments conducted in Brazil, the zebrafish were raised in the Biological Sciences Facility at the Federal University of Paraná (UFPR). All experiments complied with the Brazilian Guidelines for Care and Use of Animals for Scientific and Teaching Purposes, established by the National Council for the Control of Animal Experimentation (CONCEA), and were approved by the Ethics Committee for Animal Use from the Biological Sciences Section of UFPR (CEUA/BIO – UFPR), under protocol number 1410 (process 23075.033243/2021-99).

### Published RNAseq data analysis

A Microsoft Excel formula (see formula below, alphanumerics in bold are example cells) was used to identify mutual significantly upregulated genes in RNAseq datasets on IL-4/IL-10-polarized human MDMs and zebrafish macrophages infected with *Mm*. As only one gene was originally identified, genes upregulated in the human MDM dataset were also compared to non-significantly upregulated zebrafish genes to broaden the search.

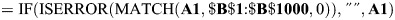
The MDM RNAseq dataset used to determine candidate genes can be accessed at doi:10.17632/j2hmt7k9fh.1 (Baidžajevas et al., 2020). Zebrafish macrophage RNAseq datasets are available at the NCBI Gene Expression Omnibus under accession numbers GSE78954 (uninfected macrophages) and GSE68920 (*Mm*-infected macrophages) (Rougeot et al., 2019). Single-cell RNAseq data on adult zebrafish leukocytes can be accessed at BASiCz – Blood Atlas of Single Cells in zebrafish (Athanasiadis et al., 2017).

### crRNA design and synthesis

The selected target genes were *ctsz* (ENSDARG00000043081), *nccrp1* (ENSDARG00000035326) and *ocstamp* (ENSDARG00000022139). Guides for targeting the zebrafish genome were designed using the online tool Custom Alt-R™ CRISPR-Cas9 guide RNA. A pair of guides was designed for each gene: one targeting the coding sequence and the other targeting the gene promoter region. Selection criteria for the sequences included proximity to the ATG start codon, localization within the first three exons, and on-target potential and off-target risk scores greater than 80.

### CRISPR/Cas9 reagents for microinjection

The Alt-R™ CRISPR-Cas9 system consists of crRNA and tracrRNA (IDT) combined with Cas9 nuclease protein (IDT), which is recombinant, purified from a strain of *Escherichia coli* and derived from *Streptococcus pyogenes*. Transactivating RNAs (tracrRNAs) and gene-specific CRISPR RNAs (crRNAs) were resuspended in nuclease-free water at a concentration of 20 μM. The sequence of guides is shown in Table 1. crRNA targeting the *tyr* gene, which encodes tyrosinase required for melanin synthesis, was used as a visual marker of editing efficiency (Jao et al., 2013); successful knockdown results in an albino phenotype without affecting inflammatory responses. Larvae were also injected with guide RNAs targeting the genes *ctsz*, *nccrp1* and *ocstamp*. The components were mixed in a 1:1:1 ratio of crRNA:tracrRNA:Cas9 protein. For guide hybridization, the crRNA and tracrRNA were first mixed and incubated at 95°C for 5 min. After incubation and cooling to room temperature, the Cas9 protein was added. To ensure complete complexation of the protein and the guide, the solution was incubated for 10 min before starting injections.

**Table 1.** Summary of the CRISPR-Cas9 guide RNAs, the location relative to the gene start codon and the size of the deletion generated

Gene	Region	Guide RNA (5′-3′)	Deletion size
*ctsz*	ATG upstream	TATAGAAGGTCATTACCCGTAGG	3185 bp
	CDS	TTCATAGACTCATAAGGACGTGG	
*nccrp1*	ATG upstream	AGTGATTCCACCTTGTGAAA TGG	5406 bp
	CDS	CAATGCCTGACACCGTGGACTGG	
*ocstamp*	ATG upstream	GAGGCCACTTCTGCAACTTGGGG	7897 bp
	CDS	CCCGACACTGTTTACCCAACAGG	
*tyr*	CDS	GGACTGGAGGACTTCTGGGGAGG	−

The three nucleotides referring to the protospacer adjacent motif sequence are underlined. CDS, coding sequence.

### Genotyping of crispant larvae

To validate and determine the efficiency of CRISPR/Cas9 in inducing gene-specific mutations, injected larvae were genotyped using PCR. Genomic DNA was extracted from individual larvae at 5 dpf. Each larva was placed in a 0.2 ml PCR tube containing 50 µl of 50 mM NaOH and heated at 95°C for 30 min in a thermocycler. 5 µl of 1 M Tris-HCl (pH 8.0) was then added to the reaction and thoroughly mixed by vortexing. 1 µl guide DNA was directly used as a template for the PCR reaction.

The PCR was conducted using FirePol^®^ DNA polymerase (Solis BioDyne) to amplify the targeted genomic region. Gene-specific primers were designed with the Primer3 online tool (Primer3). For each gene, three primers were designed to amplify a WT allele, as well as an INDEL, in which both guides have successfully caused a DNA double-strand break. The primer sequences are shown in Table S1.

The PCR products were analysed by electrophoresis on a 2% agarose gel. Briefly, DNA samples were subjected to horizontal electrophoresis on a 2% agarose gel with SYBR™ safe DNA gel stain (Invitrogen, Thermo Fisher Scientific) in the TAE (Tris-acetate-EDTA) buffer. The electrophoresis chamber was connected to a power supply, and runs were performed at 110 V for 45 min. The run time was monitored by the migration of the molecular mass marker, and, after the run, the gel was revealed on a UVP transilluminator.

The efficiency of the CRISPR system in generating mutations in the *ctsz* gene was 50.61%, because 41 of the 81 injected animals had detectable mutations in genotyping. The efficiency in generating mutations in the *nccrp1* gene was 67.64% (changes in 69 of the 102 injected animals), and in the *ocstamp* gene it was 53% (changes in 35 of the 66 injected animals) (Fig. S3).

### Induction of sterile inflammation in crispant larvae

Zebrafish were dechorionated at 3 dpf using sterile laboratory tweezers, and the larvae were subsequently anesthetized in MS-222 Tricaine solution (0.168 mg/ml; Sigma-Aldrich) prepared in E3 medium. To induce an inflammatory response, the larvae were placed under a dissecting microscope (Leica Microsystems), where the caudal fin was transected using a scalpel blade (5 mm depth; WPI). The transection was made immediately posterior to the circulatory loop, ensuring that the loop remained intact, as previously described (Renshaw et al., 2006). The larvae were then recovered and kept at 28°C in fresh E3 medium in 48-well plates.

### Caudal fin regeneration assay

Zebrafish larvae from the *TgBAC(csf1ra:eGFP)sh577* and *Tg(mpeg1.1:nls-clover/tnfα:mcherry)* lines were monitored via microscopy at different time points following caudal fin transection. A single brightfield *z*-slice image was acquired using a 20× objective on a Te2000-U Eclipse microscope (Nikon). A focused brightfield image of the circulatory loop in the tailfin was captured immediately after the injury (0 hpi) and subsequently at 24 and 48 hpi. The regenerated area was measured by drawing a line parallel to the notochord, starting from the circulatory loop and extending to the tail's distal edge. Regeneration quantification was performed using ImageJ software. The normalized regeneration rate was calculated for three intervals: regeneration during the first 24 hpi (area at 24 hpi−area at 0 hpi), regeneration during the second 24 hpi (area at 48 hpi−area at 24 hpi) and total regeneration up to 48 hpi (area at 48 hpi−area at 0 hpi).

### Generation of a *Tg(tnfα:mCherry-F)sh716* transgenic line

*tnfα:eGFP-F*- and *mfap4:mCherry-F*-containing plasmids (kind gifts from Dr Mai Nguyen-Chi, University of Montpellier, Montpellier, France) were digested using NotI-HF and SacI-HF to isolate the *tnfα* promoter (Gene ID: 405785) and farnesylated *mCherry* (Nguyen-Chi et al., 2015). Digest products were purified and ligated (T4 ligase, NEB). Whole-plasmid sequencing was performed by Plasmidsaurus using Oxford Nanopore Technology with custom analysis and annotation. Forward (5′-TTGCGGCCGCTAATTGCTGTATGTCTTAAAG-3′) and reverse (5′-ATCAACATAAAATGAATCAATG-3′) primers were designed using SnapGene software to amplify the *tnfα* promoter via PCR, removing the ATG. The PCR product was purified and digested using NotI-HF (NEB) and ligated into the *tnfα:mCherry-F* vector at NotI-HF and EcoRV-HF (NEB) sites prior to transformation to competent *E. coli* cells (NEB). DNA was extracted and purified using a Qiagen MIDIprep kit according to the manufacturer's instructions, and whole-plasmid sequencing was performed by Plasmidsaurus to check for correct insertion.

Cloned *tnfα:mCherry-F* plasmid was injected into single-cell-stage nacre embryos according to a published I-SceI meganuclease transgenesis protocol (Soroldoni et al., 2009). To ensure that expression was macrophage specific, F0 adults were outcrossed to *mpeg1:nls-clover.* Founders identified were crossed to nacre WT fish, injured and screened. Subsequent embryos were screened for mCherry expression in the pharynx using a stereo microscope, as previously described (Nguyen-Chi et al., 2015).

### Macrophage quantification assay for *nccpr1* and oc*stamp* mutants

Zebrafish larvae from the *TgBAC(csf1ra:eGFP)sh577* line were monitored using fluorescence microscopy following caudal fin transection at 24 and 48 hpi. After the larvae were embedded in 1% low-melting-point agarose with tricaine, images were acquired using a Nikon Te2000-U Eclipse inverted fluorescence microscope. GFP fluorescence was detected using a filter set with excitation at 488 nm. Images were captured with a 20× objective, using a standard exposure time of 500 ms and a Z-piezo movement of 100 µM.

Fluorescence intensity per pixel was analysed using ImageJ Fiji [National Institutes of Health (NIH)]. *Z*-stacks were compressed for maximum projection, and the region of interest for the regenerative area was delineated from the circulatory loop. Background subtraction was performed using a rolling ball radius of 50 pixels. Pixel intensity normalization was achieved by dividing the raw integrated density (RawIntDen) by the defined area. Because the pattern of GFP expression was not sufficient to delineate a single cell, the results were expressed as fluorescence intensity emitted by these cells.

### Total macrophage and pro-inflammatory macrophage assay in *ctsz* mutants

For this assay, *Tg(mpeg1:nls-clover/tnfα:mcherry)* zebrafish were used. After embedding the animals in 1% low-melting-point agarose with tricaine, images were acquired using a Nikon Te2000-U Eclipse inverted fluorescence microscope. Clover fluorescence was detected using a filter set with excitation at 488 nm, and mCherry fluorescence was detected using a filter set with excitation at 543 nm and detected in the red detection channel (550–650 nm). Both lasers were set at an intensity of 5.00. High-resolution images were captured with a configuration of one frame every 4 s and a resolution of 1024. Images were captured with a 20× objective, using a standard exposure time of 500 ms and a Z-piezo movement of 100 µM. The image capture and processing software utilized was NIS Elements 4.20 Confocal. The number of macrophages at the tailfin wound was determined by manually counting cells that expressed clover, under the *mpeg1* promoter. The number of inflammatory macrophages was determined by manually counting cells that expressed the fluorescent proteins mCherry under the *tnfα* promoter, which were co-expressed. The images were analysed using ImageJ/Fiji software (NIH). Whole-body macrophage numbers were assessed by counting the number of *mpeg1*^+^ cells in unstimulated *tyr* or *ctsz* crispants using TrackMate software (Tinevez et al., 2017).

### Statistical analysis

Statistical analyses were performed using GraphPad Prism software, applying two-way ANOVA with Bonferroni or Šidák multiple comparisons tests.

## Supplementary Material

10.1242/dmm.052520_sup1 Supplementary information
