# Supplementary material for: Loss of Cathepsin Z enhances pro-inflammatory macrophage responses and promotes tissue regeneration
Source: Dis Model Mech. 2026 Apr 29;19(4):dmm052520. doi: 10.1242/dmm.052520 (PMC13225230; doi:10.1242/dmm.052520)
Supplement: Supplementary information [file dmm-19-052520-s1.pdf]

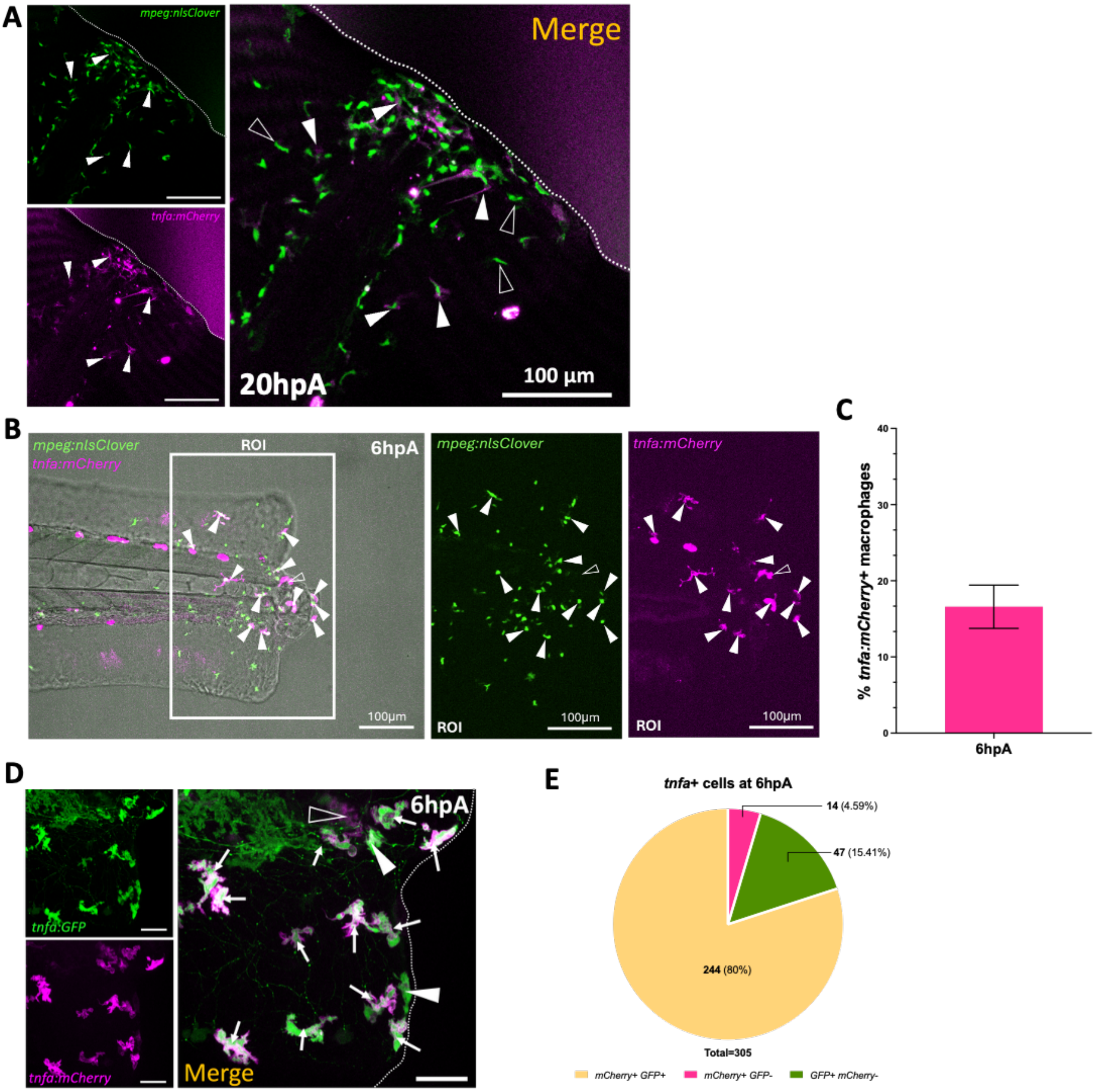

Fig. S1. A specific subset of macrophages upregulate tnfa:mCherry following tail fin transection. (A) Representative confocal image illustrating co-localisation of tnfa:mCherry+ mpeg:nlsClover+ macrophages at the injury site at 20 hpA. Hollow arrowheads indicate tnfa:mCherry- mpeg:nlsClover+ cells. Filled arrowheads indicate tnfa:mCherry+ mpeg:nlsClover+ cells. **(B)** Representative frame (maximum projection) of a timelapse of a

Disease Models & Mechanisms • Supplementary information

tnfa:mCherry+ mpeg:nlsClover+ larvae at 6 hpA. White ROI box indicates the area of the wound site where macrophages were quantified, with ROI area in the GFP and mCherry channels illustrated to the right. Filled arrowheads indicate tnfa:mCherry+ mpeg:nlsClover+ macrophages. Hollow arrowhead indicates a tnfa:mCherry+ mpeg:nlsClover- cell. **(C)** Graph illustrating 20% of tnfa:mCherry+ macrophages identified in the ROI at 6 hpA, in-line with previously published work characterising the Tg(tnfa:GFP-F) promoter (Nguyen-Chi et al., 2015). Error bars depict SEM. Timelapse time steps were 3 minutes. N=6 larvae from 1 independent experiment. **(D)** Representative confocal image of the tail fin of a tnfa:GFP+ tnfa:mCherry+ fish at 6 hpA. **(E)** A pie chart illustrating that the TgBAC(tnfa:GFP) (Marjoram et al., 2015) and Tg(tnfa:mCherry-F) transgenic lines have similar expression patterns following a tail fin injury. Scale bars in (A) and (B) are stated on the images. Scale bar in (D) is 100μm

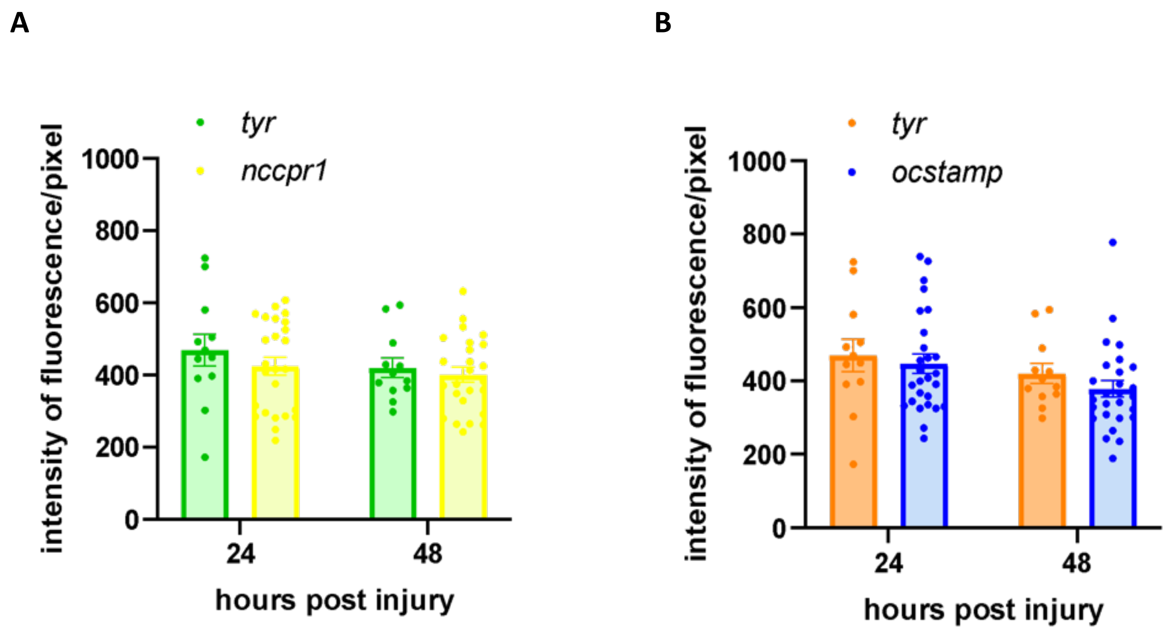

Fig. S2. Quantification of fluorescence intensity in macrophages *tyrosinase* control and crispant for the *nccrp1* and *ocstamp* genes, at 24- and 48- hpi. Transgenic *Tg*BAC(csf1ra:eGFP)sh377 animals were injured, and each point corresponds to a single larva. **(A)** Quantification of fluorescence intensity in crispant for *nccrp1* crispant (yellow) and *tyr* (green), n = 12-21, pooled from three independent repeats. **(B)** Quantification of fluorescence intensity in *ocstamp* crispant (blue) and *tyr* (orange), n = 12-27, pooled from three independent repeats. The analysis was performed using a two-way ANOVA with Bonferroni multiple comparison test; error bars represent the mean ± SD. hpi = hours post-injury.

Disease Models & Mechanisms • Supplementary information

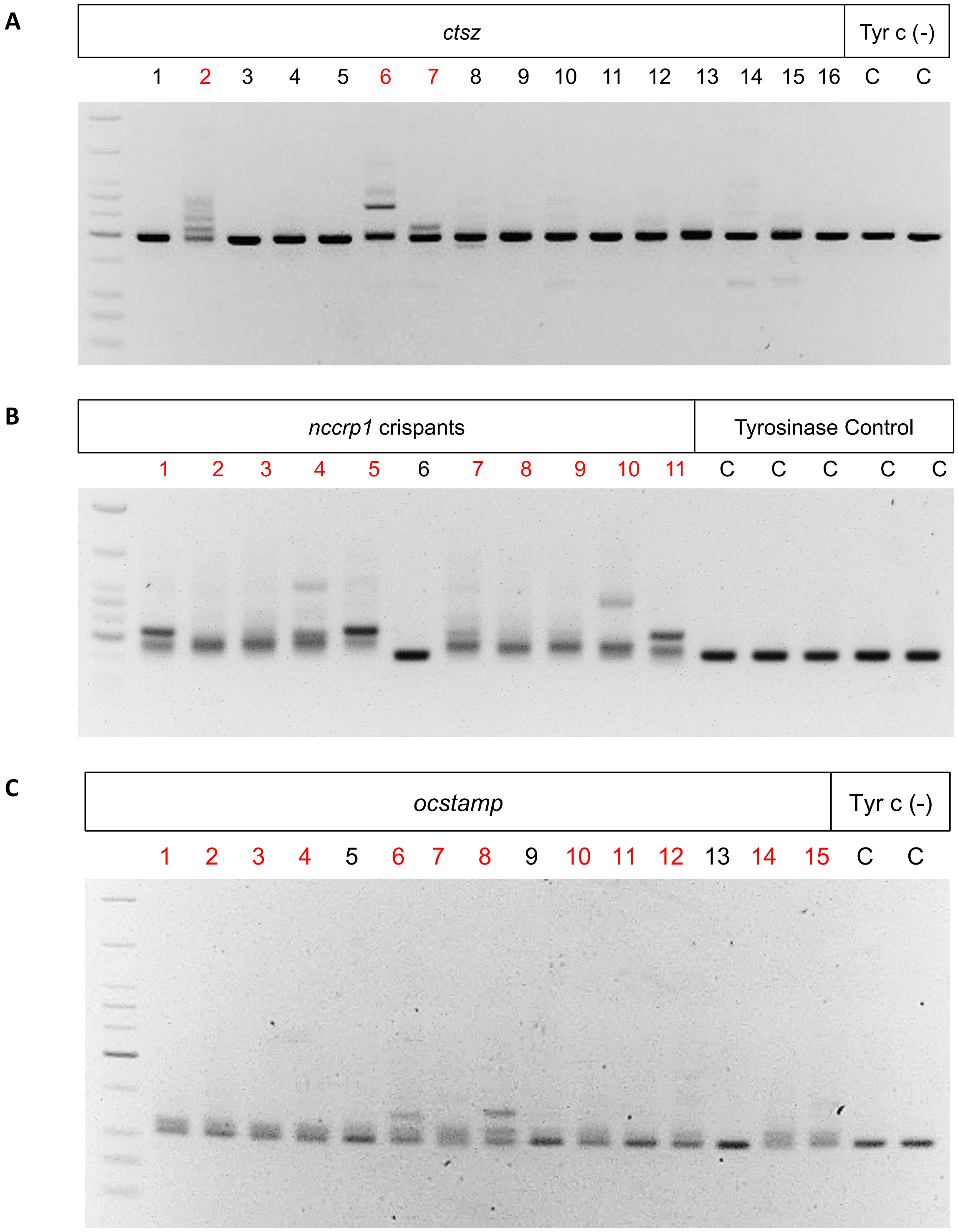

Fig. S3. Genotyping electrophoresis of animals injected with both sgRNAs targeting the promoter region and CDS of the gene, followed by *tyr* control samples. The numbers of animals carrying INDELs are highlighted in red, and control animals are identified with the

letter C. Agarose gel electrophoresis (2%) was performed for 45 minutes at 120 V. The first well (LMW) was loaded with 5 µL of Quick-Load® Purple Low Molecular Weight DNA Ladder (NEB, UK). DNA fragments were visualised using a U:GENIUS3 transilluminator (Syngene®, UK). **(A)** Genotyping of 16 animals injected with *ctsz* sgRNAs, followed by 2 *tyr* control animals. **(B)** Genotyping of 11 animals injected with *nccrp1* sgRNAs, followed by 4 *tyr* control animals. **(C)** Genotyping of 15 animals injected with *ocstamp* sgRNAs, followed by 2 *tyr* control animals.

Table S1. Primer sequences.

	**Sequence 5' – 3'**
fw_1-ctsz	CAT GAA AAC TGC TCT AAA AGG GT
rev_2- ctsz	CCT TTC CCC TCA CCT GCT AA
fw_3- ctsz	ACT TTA TGC GTT TTA CAG GAC TG
fw_1-nccrp1	TCT AAC TGC AGT CTA GCC GC
rev_2- nccrp1	GAA TAC GCC TGT GTG TGA CG
fw_3- nccrp1	AGC AGA AGT GTG ACT CGG AA
fw_1- ocstamp	AAC TGG TGG ATT CCT GCT GT
rev_2- ocstamp	GGA CGT ACA GCA CCA CAA TC
fw_3- ocstamp	ATC CAT TCC GAT GTG CCG T

Disease Models & Mechanisms • Supplementary information
